# “GOD ALONE EXISTS IN SOLITUDE”: INTERVIEWING OLD MIGRANTS ABOUT LONELINESS

**DOI:** 10.1093/geroni/igad104.1313

**Published:** 2023-12-21

**Authors:** Basak Bilecen

**Affiliations:** University of Groningen, Groningen, Groningen, Netherlands

## Abstract

Current research emphasizes the need for a more critical reflective approach to studying older migrants, with particular attention paid to methodological and ethical challenges during fieldwork. One of the key methodological challenges researchers face is selecting appropriate questions, as the questions asked can influence the insights gained into the phenomenon under study. This is because researchers gain an understanding of the phenomenon based on the questions they ask. Previous research on the well-being of older migrants has primarily focused on their family relationships, health status, social participation into activities, and vulnerabilities. However, an important question to understand the well-being of older migrants is whether and to what extent they experience loneliness. This study involved conducting 20 semi-structured interviews with first-generation Turkish migrants over the age of 65 living in northern Netherlands. The findings suggest that the phrasing of questions plays a significant role in both how participants interpret the questions and their responses. During the interviews, while loneliness was generally considered a negative issue, some participants approached it from a religious perspective, leading to reflection on more fundamental existential questions, which in turn led to denial.

